# The role of previous streptococcal infections in adult patients with obsessive-compulsive disorder: a research study

**DOI:** 10.1017/S1092852925000203

**Published:** 2025-03-24

**Authors:** Donatella Marazziti, Stefania Palermo, Alessandro Arone, Manuel Glauco Carbone, Lucia Massa, Lara Foresi Crowther, Nicola Schulz Bizzozzero Crivelli, Riccardo Gurrieri, Francesco Weiss, Federico Mucci, Liliana Dell’Osso

**Affiliations:** 1 Department of Clinical and Experimental Medicine, Section of Psychiatry, University of Pisa, Pisa, Italy; 2 Saint Camillus International University of Health and Medical Sciences, Rome, Italy; 3 Department of Medicine and Surgery, Division of Psychiatry, University of Insubria, Varese, Italy

**Keywords:** obsessive-compulsive disorder, immune system, autoimmunity, inflammation, streptococcal infection, antistreptolysin O titer

## Abstract

**Introduction:**

Autoimmune processes have been documented in both childhood and adulthood patients with obsessive-compulsive disorder (OCD), with the pediatric autoimmune neuropsychiatric disorders associated with streptococcal infections (PANDAS) representing the paradigm of this model.

Given the limited information available, the present study aimed at assessing the characteristics of adult patients with OCD exposed to a previous group A β-hemolytic Streptococcus infection, together with some peripheral inflammatory biomarkers.

**Materials and methods:**

Fifty-two subjects displaying antistreptolysin O (ASO) titer positivity were recruited from a sample of 247 adult OCD outpatients, diagnosed according to DSM-5 criteria and assessed by the Yale-Brown Obsessive-Compulsive Scale. Their clinical features were assessed and compared. The possible relationships between the different parameters were also examined.

**Results:**

Thirty-six subjects who were on medication for OCD showed significantly lower ASO titers than the other. The neutrophil count was positively and negatively related to, respectively, the “distress associated with obsessive thoughts” item and to the patients’ age. The lymphocyte count and folic acid levels were higher in 30 subjects with no perinatal insults.

**Conclusions:**

These results seem to suggest that OCD subjects with ASO titer-positivity show a chronic inflammatory state, in spite of no symptoms or recall of bacterial infections, that might be involved in both the onset and the maintenance of OCD, with immunological alterations being related to symptom dimension to be identified. They also support the notion of possible anti-inflammatory effects of some psychotropic compounds.

## Introduction

In the last years, a large amount of research has been directed towards detecting immunological alterations in both childhood and adulthood obsessive-compulsive disorder (OCD), a common and disabling psychiatric condition.^1^^–^^10^ As a result, different immunological processes have been highlighted or hypothesized in OCD while investigating the possible role of a wide range of mediators.^1^^,^^5^^,^^11^^–^^22^

The first hypothesis was formulated in 1989 with the description of a childhood syndrome related to *Streptococcus pyogenes*, a group A-streptococcus (GABHS) infection, called (PANDAS), an acronym for pediatric autoimmune neuropsychiatric disorders associated with streptococcal infections.^23^ *S. pyogenes* can cause relatively mild illness (sore throat, otitis media, sinusitis, and impetigo), or more severe and invasive conditions like necrotizing fasciitis, bacteremia, and toxic shock syndrome, and also rheumatic fever (RF) and Sydenham chorea (SC) that are due to antibody cross-reactivity with, respectively, kidney, heart, and brain antigens.^24^^–^^26^ Swedo et al. (1989) reported a higher prevalence of obsessive-compulsive symptoms (OCS) in children and adolescents with SC, in comparison to those who had RF without chorea, and proposed the existence of a link between OCD and basal ganglia dysfunction. Interestingly, the temporal association with *S. pyogenes* infection led to the hypothesis of a possible autoimmune pathogenetic mechanism similar to that characterizing SC (ie the main neurological manifestation of RF), in which streptococcal antibodies cross-react against brain antigens due to a molecular mimicry process.^27^^–^^29^

Almost 10 years later, the same group described the clinical characteristics of a new subgroup of 50 patients presenting OCD and tic disorders and meeting five diagnostic criteria: (1) presence of OCD and/or a tic disorder, (2) prepubertal onset, (3) episodic course of symptom severity, that is to say an acute symptom onset and relapsing–remitting course, (4) association with GABHS infections, and (5) association with neurological abnormalities.^30^

In 2012, PANDAS criteria were again modified to describe an expanded clinical entity, the pediatric acute-onset neuropsychiatric syndrome (PANS), characterized by abrupt, dramatic onset of OCD or severely restricted food intake and by the concurrent presence of additional neuropsychiatric symptoms with similarly severe and acute onset, while entailing that several agents other than Streptococcus, including even environmental and metabolic factors, might be involved in the pathophysiology of such clinical pictures.^31^^–^^34^

If it is true that PANDAS classically represents a pediatric syndrome, it is worth underlining that some case reports of PANDAS-like syndromes have also been reported in adults, specifically in males who developed OCD and related conditions with a temporal relation with streptococcal infections.^35^^–^^37^ Interestingly, regarding new cases of neuropsychiatric disorders in adults, some authors suggested that measuring the antistreptolysin O (ASO) titer-positivity, an extracellular antigen produced by *S. pyogenes* might represent a useful diagnostic tool from the etiologic point of view.^38^^,^^39^

Therefore, although the existence of PANDAS/PANS as a distinct entity has been debated, and its exact recognition has not met a general consensus,^33^ nonetheless, since its first postulation, it has been representing the paradigm of an autoimmune model for OCD. As such, it has promoted the evaluation of inflammatory, infective, immunologic, and metabolic alterations in patients with OCD and OCS, as well as the evaluation of antibiotics and non-steroidal anti-inflammatory drugs (NSAIDs) as novel and adjunctive treatment options.^33^^,^^40^^–^^42^ Not surprisingly, the immuno-inflammatory model of OCD appears to be a complex paradigm of intertwining factors and processes including, amongst others, blood cells, cytokines, anti-basal ganglia antibodies (ABGA) and autoimmune processes, microglia, fetal-maternal immune interactions, and the gut-brain axis.^9^^,^^10^^,^^28^^,^^43^^–^^56^ In any case, authors also suggested the possible existence of a specific “autoimmune OCD” subtype.^10^^,^^42^^,^^44^^,^^57^ Again, more recently, onset or worsening of neuropsychiatric symptoms after SARS-CoV-2 infections, including PANDAS symptoms in some case reports have been described,^58^^–^^60^ while promoting an increasing interest in infective agents that might target the central nervous system (CNS).^61^^,^^62^

Given the paucity of available data on the possible impact of GABHS infection in adult patients with OCD, the present study aimed at assessing the clinical features of patients showing ASO-titer positivity, together with a few peripheral inflammatory biomarkers, specifically white blood cell (WBC) neutrophil, lymphocyte, monocyte, and platelet counts, C-reactive protein (C-RP) and erythrocyte sedimentation rate (ESR), vitamin B12, vitamin D, folic acid, and homocysteine.

## Materials and methods

A sample of 52 (M = 33, F = 19) subjects displaying ASO titer-positivity were recruited from a total of 247 adult OCD outpatients referring to the Psychiatric Unit of Pisa in the years 2022–2023. No patients showed current symptoms of pharyngo-tonsillitis.

Gender distribution, age of the sample, age and type of OCD onset, eventual onset in relation to stressful life events (LE) or romantic relationships, course of illness, presence of obsessive-compulsive (OC) personality traits, experience of perinatal insults, marital status, education, employment, psychiatric comorbidities and presence of other medical conditions, history of substance use, past and current psychotropic treatments and psychological interventions were investigated in all patients.

Patients who were pregnant, with drug intoxication, with severe comorbid psychotic disorders or major medical illness, or suffering from cognitive impairment or dementia were excluded. All information regarding socio-demographic variables, psychiatric and medical comorbidities, and pharmacological treatment was derived from the medical history collected during the interview. After a complete description of the study, written informed consent was obtained from each subject to participate in the study.

### Psychopathological assessment

The diagnosis of OCD was carried out according to DSM-5 criteria and the Structured Clinical Interview for DSM-5, Research Version (SCID-5-RV).^63^ The Y-BOCS^64^ was used to assess the OCD severity and symptomatology. The presence of depression, as assessed by the Hamilton Depression Rating Scale (HDRS or HAM-D),^65^ represented an exclusion criterion.

#### Yale-Brown Obsessive-Compulsive Scale (Y-BOCS)

The Y-BOCS is a clinician-administered instrument to assess the presence and severity of OCS. Items are scored on a five-step Likert scale, ranging from 0 to 4, with higher scores indicating more severe symptoms.^64^ The first 10 items (1–5 for obsessions and 6–10 for compulsions) represent the core component of the Y-BOCS and assess time, interference, distress, resistance, and degree of control over OCS. The total score is obtained from the sum of the first 10 items, excluding items 1.1 and 6.1, and it can go from 0 to 40. A score of 0–7 indicates subclinical, 8–15 mild, 16–23 moderate, 24–31 severe, and 32–40 extreme symptoms.^66^ Furthermore, 9 additional items are available to assess insight, avoidance, degree of indecisiveness, overvalued sense of responsibility, pervasive slowness, pathological doubting, global severity, global improvement, and reliability.

#### Structured Clinical Interview for DSM-5 (SCID-5)

The Structured Clinical Interview for DSM-5 (SCID-5) is a clinician-administered, semi-structured interview guide built to make the major DSM-5 diagnoses.^63^ It is the most comprehensive version of the SCID-5 and contains 12 modules (A–L) mirroring the structure of the DSM-5, as well as specifiers. For example, the assessment of OCRDs (Module G) starts with three screening questions tailored to screen the various types of obsessions (ie thoughts, images, and urges), and the level of insight is assessed, as a specifier, for OCD, hoarding disorder, and body dysmorphic disorder.

#### Hamilton Depression Rating Scale (HAM-D/HDRS)

The HAM-D/HDRS^65^ is a widely used hetero-administered scale, consisting of 21 items, for the assessment of depression and its severity. The total score is based on the first 17 items. Depressed mood, feelings of guilt, suicide, initial insomnia, insomnia during the night, delayed insomnia, work and interests, retardation, agitation, psychiatric anxiety, somatic anxiety, gastrointestinal somatic symptoms, general somatic symptoms, genital symptoms, hypochondriasis, weight loss, and insight represent the 17 core items. Additional items are available to investigate diurnal variation, depersonalization/derealization, paranoid symptoms, and OCS.^67^ A score ≤ 7 indicates no depression, 8–17 mild depression, 18–24 moderate depression, and ≥ 25 severe depression.

### Biological assessment

The ASO was evaluated with an immune-turbidimetry method, and its positivity was defined by a value higher than 200 UI/mL. Common clinical chemical methods were used to assess vitamin B12, vitamin D, folate, homocysteine, and inflammatory markers. Standard serum B12 assays quantify both the inactive and active forms of serum cobalamin and are based on intrinsic factor binding and immune-chemiluminescence-based techniques. Standards of reference of B12 plasma normal levels as well as its deficiency are not well established. However, it has been proposed that serum B12 of <148 pmol/L (200 ng/L) would have the sensitivity for diagnosing 97% of true cobalamin deficiencies. Laboratories use different reference ranges and units of measurement (pmol/L or ng/L) in the absence of a standardized methodology.^68^ Competitive folate binding protein assays using chemiluminescence or fluorescence detection systems are the techniques most frequently used by laboratories to measure folate plasma levels. Although there is no consensus on folate plasma levels indicative of folate deficiency, usually levels <7 ng/mL (3 μg/L) are taken as reference since they are associated with a strong increase in risk for megaloblastic anemia, while the significance of low levels, between 7 and 10 nmol/L (3 and 4–5 μg/L), is still unclear.^68^ To conduct this analysis, as suggested by the World Health Organization (WHO), we took as reference the levels of 10 nmol/L (4 ng/mL) and of <150 pmol/L (203 pg/mL) as indicative of, respectively, folate and B12 deficiencies.^69^ 25(OH)D concentrations were used as a marker of vitamin D status measurements and assessed by common clinical chemical methods. It was considered optimal for levels >30 ng/mL, sufficient between 20 and 30 ng/mL, insufficient for 12–20 ng/mL, deficient for 6–12 ng/mL, and severely deficient for <6 ng/mL.

Vitamin D was assessed in 49 (M = 32, F = 17), folate in 42 (M = 29, F = 13), and vitamin B12 in 38 (M = 25, F = 13) patients. White blood cell neutrophil, lymphocyte, monocyte, and platelet counts were measured in 32 (M = 25, F = 7) patients. Homocysteine levels were assessed in 18 (M = 12, F = 6) subjects. Both C-RP and ESR values were measured in 31 (M = 21, F = 10) patients. Regarding blood exams, the cut-offs for ESR and C-RP positivity were, respectively, >20 mm/h and > 6 mg/mL.

### Statistical analyses

All demographic, clinical, and laboratory data were presented for continuous variables in terms of mean ± standard deviation (SD) and variation range (min and max values) when required. Categorical variables were expressed as frequencies (number) and percentages.

The Kolmogorov–Smirnov test was used to determine the normality of the distribution of the variables.

Comparisons for continuous variables were performed with the independent-sample Student’s t-test for variables that follow a normal distribution, Wilcoxon–Mann–Whitney test for variables not normally distributed.

The correlations were explored by calculating the Pearson’s correlation coefficient or Spearman’s rank correlation. Pearson’s r correlation is used to measure the degree of the relationship between linearly related variables. For the Pearson’s r correlation, both variables should be normally distributed (normally distributed variables have a bell-shaped curve). Other assumptions include linearity and homoscedasticity. Linearity assumes a straight-line relationship between each of the two variables, and homoscedasticity assumes that data is equally distributed about the regression line. Spearman’s rank correlation is a non-parametric test that is used to measure the degree of association between two variables. Spearman rank correlation test does not carry any assumptions about the distribution of the data and is the appropriate correlation analysis when the variables are measured on a scale that is at least ordinal. The assumptions of the Spearman correlation are that data must be at least ordinal and the scores on one variable must be monotonically related to the other variable.

The major limitation of the present study is the low number of subjects selected. Due to the high possibility of both type I and type II errors, our results should be considered preliminary. In this study, p-values lower than .05 were considered statistically significant.

Statistical analysis was performed using SPSS 25.0 software (IBM Corp., Armonk, NY, USA).

## Results

### Socio-demographic characteristics

Half of patients (26) experienced an onset of the disorder in relation to a stressful LE, while 8 (15.4%) to the end of a romantic relationship.

Thirty-five (67.3%) and 17 (32.7%) patients had, respectively, a progressive and a sudden-onset. Forty-six (88.5%) and 6 (11.5%) patients had, respectively, a chronic and an episodic OCD. Twenty-two (42.3%) male patients, out of the total 33, had experienced perinatal/obstetric insults, while none of the 19 females reported this occurrence. Twenty-eight (53.8%) subjects showed OC personality traits (Table 1).

**Table 1.** Socio-demographic Characteristics of Outpatients with OCD

		Total (n = 52)	Male (n = 33)	Female (n = 19)
Mean age at onset ± SD		16.4 ± 6.5	15.8 ± 6.1	17.5 ± 7.1
Course	Episodic	6 (11.5%)	3 (9.1%)	3 (15.8%)
	Chronic	46 (88.5%)	30 (90.9%)	16 (84.2%)
Onset	Sudden	17 (32.7%)	10 (30.3%)	7 (36.8%)
	Progressive	35 (67.3%)	23 (69.7%)	12 (63.2%)
Onset related to stressful life events		26 (50.0%)	16 (48.5%)	10 (52.6%)
Onset related to the end of a relationship		8 (15.4%)	5 (15.2%)	3 (15.8%)
Perinatal insults		22 (42.3%)	22 (66.7%)	0
Obsessive-compulsive personality traits		28 (53.8%)	20 (60.6%)	8 (42.1%)
Education level	University Degree	17 (32.7%)	11 (33.3%)	6 (31.6%)
	High school	31 (59.6%)	19 (57.6%)	12 (63.2%)
	Middle school	4 (7.7%)	3 (9.1%)	1 (5.3%)

In terms of comorbidity, 10 patients had bipolar disorder, 6 panic disorder, 3 Tourette’s disorder (TS), 2 autism-spectrum disorder, 1 generalized anxiety disorder, 1 alcohol use disorder, and 1 eating disorder.

Thirty-four (65.4%) patients had previously taken psychopharmacological treatments, and 26 (50%) had undergone Cognitive Behavioral Therapy (CBT). At the moment of the observation, 36 (69.2%) patients were taking psychotropic drugs, while 16 (30.8%) were drug-free. Eight (15.4%) subjects were undergoing CBT.

### Clinical characteristics

The Y-BOCS total score (mean ± SD) was 25.5 ± 6.19, and the obsession and compulsion scale scores (mean ± SD) were, respectively, 12.79 ± 3.13 and 12.90 ± 3.29. The most common current obsessions were “other” (n = 41; 78.8%), “aggressive” (n = 36; 69.2%), “symmetry” (n = 28; 53.8%), “contamination” (n = 21; 40.4%), “somatic” (n = 14; 26.9%), “religious” (n = 11; 21.2%), “hoarding” (n = 9; 17.3%), and “sexual” (n = 7; 13.5%). Past obsessions were represented as follows: 39 (75%) subjects reported “other,” 34 (65.4%) “aggressive,” 26 (50%) “symmetry,” 21 (40.4%) “contamination,” 14 (26.9%) “somatic,” 11 (21.2%) “religious,” 9 (17.3%) “hoarding,” and 4 (7.7%) “sexual” obsessions. The most common current compulsions were “control” (n = 44; 84.6%), “other” (n = 34; 65.4%), “repetitive rituals” (n = 25; 48.1%), “cleaning/washing” (n = 24; 46.2%), “counting” (n = 13; 25%), and “hoarding.”(n = 5; 9.6%). As regards past compulsions, 43 (82.7%) patients reported “control,” 35 (67.3%) “other,” 27 (51.9%) “repetitive rituals,” 24 (46.2%) “cleaning/washing,” 13 (25%) “counting” and 4 (7.7%) “hoarding” compulsions. Insight was distributed as follows: 36 (69.2%) patients scored 0, 11 (21.2%) scored 1, 3 (5.8%) scored 2, and 2 (3.8%) scored 3.

### Laboratory tests

The ASO titer (IU/mL, mean ± SD) was 426.80 ± 152.05 (range: 232–916). Folate levels (nmol/L, mean ± SD) were 4.60 ± 2.23 (range: 1.20–9.30): a folate deficiency was found in 20 (M = 17, F = 3) out of the 42 subjects analyzed. The B12 levels (pmol/L, mean ± SD), measured in 38 patients, were 434.19 ± 179.43 (range: 84–792): a deficit of vitamin B12 was detected in 4 (M = 2, F = 2) out of the 38 patients who were assessed for this vitamin. Vitamin D levels (ng/mL, mean ± SD) were 19.05 ± 11.55 (range: 5.00–45.55) in the total 49 subjects, with a deficiency assessed in 39 (M = 27, F = 12) (Table 2).

**Table 2.** ASO Titer, Folate, Vitamin D, and Vitamin B12 Levels in Outpatients with OCD

	N	Min. value	Max. value	Mean ± SD	Positivity or deficiency	
ASO titer (IU/mL)	52	232.00	916.00	426.80 ± 152.05	Total	52 (100.0%)
					Male (n = 33)	33 (100.0%)
					Female (n = 19)	19 (100.0%)
Folate (nmol/L)	42	1.20	9.30	4.60 ± 2.23	Total	20 (47.6%)
					Male (n = 29)	17 (58.6%)
					Female (n = 13)	3 (23.1%)
Vitamin D (ng/mL)	49	5.00	45.55	19.05 ± 11.55	Total	39 (79.6%)
					Male (n = 32)	27 (84.4%)
					Female (n = 17)	12 (70.6%)
Vitamin B12 (pmol/L)	38	84.00	792.00	434.19 ± 179.43	Total	4 (10.5%)
					Male (n = 25)	2 (8.0%)
					Female (n = 13)	2 (15.4%)

### Intergroup differences

Married subjects had a statistically significant higher age of onset (Z = −2.903, p = .004). Employed subjects had statistically significant lower ESR (Z = −2.362, p = .018).

Thirty-six patients who were taking pharmacological treatment at the moment of assessment showed significantly lower ASO titer levels (Z = −1.999, p = .046).

Patients with the OCD onset related to stressful LE or to a romantic relationship had, respectively, statistically significant lower levels of vitamin B12 (Z = −1.981, p = .048) and lower levels of homocysteine (Z = −2.183, p = .029). A progressive onset OCD, in comparison with a sudden one, was associated with a statistically significant higher platelet count (Z = −2.800, p = .005).

Subjects who experienced no perinatal insults had significantly higher folate levels (Z = −2.382, p = .017).

Past and current contamination obsessions were related to significantly lower platelet count (Z = −2.093, p = .036) (Z = −2.431, p = .015). Folate deficit was associated with a statistically significant lower monocyte count (Z = −2.290, p = .022). Subjects with normal vitamin B12 levels showed significantly higher WBC count (Z = −1.990, p = .047) and higher ESR (Z = −2.153, p = .031). Subjects with vitamin D deficiency had a significantly higher BMI in comparison with subjects who had normal levels (Z = −3.895, p < .001).

Five (9.6%) patients who were taking lithium, in comparison to those not taking it, showed higher ASO titer levels (Z = −2.359, p = .018) and lower monocyte count (Z = −2.153, p = .031): However, given the small number of subjects, this finding should be considered just a trend.

When the patients were analyzed according to the sex, it emerged that women showed statistically significantly higher folate levels (Z = −2.369, p = .018) (Table 3).

**Table 3.** Intergroup Differences between Different Variables

Age of onset (years)	Married: 9 (17.3%)	Single: 43 (82.7%)	0.004
Erythrocyte sedimentation rate	Unemployed: 22 (42.3%)	Employed: 30 (57.7%)	0.018
Erythrocyte sedimentation rate	Vit B12 deficiency: 4 (7.7%)	Normal Vit B12 levels: 48 (92.3%)	0.031
Vitamin B12 levels	Onset related to stressful life events n = 26 (50.0%)	No relation with stressful life events: 26 (50.0%)	0.048
Homocysteine levels	Onset related to the end of a relationship n = 8 (15.4%)	Onset unrelated to the end of a relationship: 44 (84.6%)	0.029
Folate levels	Perinatal insults: 22 (42.3%)	No perinatal insults: 3 (52.7%)	0.017
Folate levels	Female: 19 (36.5%)	Male: 33 (63.5%)	0.018
Y-BOCS obsession subscale score	University degree: 17 (32.7%)	Middle school: 4 (7.7%)	0.031
Y-BOCS compulsions subscale score	University degree: 17 (32.7%)	High school: 31 (59.6%)	0.003
ASO titer	On treatment: 50 (96.2%)	Not in treatment: 2 (3.8%)	0.046
ASO titer	On lithium: 5 (9.6%)	Not on lithium: 47 (90.4%)	0.018
Monocyte count	On lithium: 5 (9.6%)	Not on lithium: 4 (90.4%)	0.031
Monocyte count	Folate deficit: 20 (38.5%)	No folate deficit: 32 (62.5%)	0.022
Platelet count	Progressive onset: 35 (67.3%)	Acute onset: n = 17 (32.7%)	0.005
Platelet count	LT contamination obs n = 21 (40.4%)	No LT contamination obs n = 31 (59.6%)	0.036
Platelet count	Current contamination obs n = 21 (40.4%)	No current contamination obs n = 31 (59.6%)	0.015
WBC count	Vit B12 deficiency: 4 (7.7%)	Normal Vit b12: 48 (92.3%)	0.047
BMI	Vit D deficiency: n = 39 (75%)	No Vit D deficiency n = 10(19.23%)	<0.001

### Non-parametric correlations

The ASO titer showed a positive correlation with vitamin B12 levels (Rho = .399, p = .013) and WBC counts (Rho = .360, p = .043). Patients’ age and age of OCD onset were inversely related to, respectively, the number of neutrophils (Rho = −.403, p = .027) and both lymphocyte count (Rho = −.492, p = .006) and homocysteine values (Rho = −.583, p = .011). Folate levels were directly associated with the “degree of control over obsessions” item scores (Rho = −.310, p = .046) and inversely with monocyte count (Rho = .579, p = .002). Vitamin B12 levels were negatively correlated with “overvalued sense of responsibility” (Rho = −.431, p = .007) and positively with the “reliability” item scores (Rho = .322, p = .049), WBC count (Rho = .490, p = .011), and monocyte count (Rho = .579, p = .002). The WBC count was negatively related to “avoidance” (Rho = −.394, p = .026) and “overvalued sense of responsibility” (Rho = −.371, p = .037) item scores and directly to neutrophil counts (Rho = .485, p = .007). The number of neutrophils was negatively related to “distress associated with obsessions” item scores (Rho = −.438, p = .016). The latter was also directly related to the BMI (Rho = .451, p = .016). C-RP values were negatively associated with the “reliability” item score (Rho = −.357, p = .049). Platelet count was negatively correlated to “reliability” (Rho = −.410, p = .022) and “insight” (Rho = −.453, p = .010) item scores (Table 4).

**Table 4.** Correlations between Different Variables

		Rho	p
Vitamin B12 levels	Reliability	0.322	0.049
Vitamin B12 levels	Overvalued sense of responsibility	−0.431	0.007
Folate levels	Monocyte count	0.579	0.002
Folate levels	Degree of control over obsessions	−0.310	0.046
Age of onset	Lymphocyte count	−0.492	0.006
Age of onset	Homocysteine levels	−0.583	0.011
Age	Neutrophils count	− 0.403	0.027
ASO titer levels	Vitamin B12 levels	0.399	0.013
ASO titer levels	WBC count	0.360	0.043
WBC count	Vitamin B12 levels	0.490	0.011
WBC count	Avoidance	−0.394	0.026
WBC count	Overvalued sense of responsibility	−0.371	0.037
WBC count	Neutrophils count	0.485	0.007
Monocyte count	Vitamin B12 levels	0.579	0.002
Neutrophils count	Distress associated with obsessions	−0.438	0.016
BMI	Distress associated with obsessions	0.451	0.016
C-RP	Reliability	−0.357	0.049
Platelet count	Reliability	−0.410	0.0222
Platelet count	Insight	−0.453	0.010

## Discussion

The aim of the present study was to investigating the clinical features of OCD subjects who had been exposed to GABHS infection in the past, as assessed by ASO titer-positivity, although with no symptoms of pharyngotonsillitis or other related symptoms. Nonetheless, from a lifetime perspective, all patients had been asymptomatic for streptococcal infection. Therefore, it should be underlined that our sample of OCD patients differs from those without ASO titer-positivity and also from both children and adults diagnosed with PANDAS, as they had no past or current symptoms of infections. The overall prevalence of ASO positivity was about 25% of the total OCD patients recruited in 2 years in our department, while suggesting that this silent infection is quite common and perhaps more than fortuitous. According to our knowledge, this is the first study of this kind, as previously only OCD onset following diagnosed streptococcal infections was reported in adult patients.^35^^–^^37^

The mean Y-BOCS score (25.5) of our sample indicates a general severe OCD clinical picture. The mean age at onset was 16.44 years, which is about 10 years later than that observed in sudden early-onset OCD occurring in children with GABHS-related neuropsychiatric syndromes. Indeed, only 32.7% of our sample had experienced a sudden onset, so again, we underline that no patients recalled a past history of pharyngotonsillitis.

Therefore, our next step was to describe possible specific features of this group of patients, as well as patterns of immune alterations. As hypothesized, male subjects represented the majority of our sample (M = 33; 63.5%): this is consistent with previous data gathered in children and adolescents with PANDAS.^70^^,^^71^ Further, one third of our male patients (22 out of 33) had suffered from perinatal insults or obstetric complications. This is not surprising, as males seem to be more vulnerable to insults of different kinds with a detrimental effect on the CNS if occurring in specific periods of life (birth, childhood, and adolescence). Interestingly, pathophysiological mechanisms in OCD seem to differ by sex,^72^ and a dimorphic pattern of genetic susceptibility has been hypothesized that would contribute to the heterogeneity of OCD from a biological and clinical point of view.^73^ Furthermore, a study conducted in OCD adult patients reported that maternal edema during pregnancy and prolonged labor were possibly associated with the expression of OCD later in life.^29^ The present study seems to confirm the detrimental role of perinatal complications for the development of OCD also in adults and in subjects without comorbidities in the area of neurodevelopmental disorders.

In terms of specific symptoms, the most represented past and current obsessions were “other” (ie miscellaneous obsessions), followed by the “aggressive” ones. The most common past and current compulsions were the “control” ones, followed by miscellaneous compulsions. These symptoms are quite different from those reported in childhood PANDAS, where the most frequent are need for symmetry and exactness, bedtime rituals, counting, and repeating compulsions.^32^^,^^74^

The subjects who were on regular medication for OCD at the time of assessment had significantly lower ASO titers than the drug-free patients, while highlighting the potential anti-inflammatory role of psychotropic compounds.^75^^–^^78^ The ASO titers were positively related to current lithium treatment and the number of WBCs, although in five patients only, so that it should be considered a mere suggestion. However, this finding, if corroborated in the larger samples, might be consistent with the notion that lithium could increase a positive inflammatory response,^79^ or might indicate the need of lithium prescription in those patients that showed a worse global clinical picture, possibly sustained by a systemic inflammation, at least as demonstrated by the higher ASO titers.

A higher WBC count was positively related to the Y-BOCS “overvalued sense of responsibility” and “avoidance” item scores. The neutrophil count and the BMI had, respectively, a positive and a negative correlation with the “distress associated with obsessive thoughts” item score. The number of neutrophils was also inversely correlated with the patients’ age, while the lymphocyte count and the levels of homocysteine showed negative correlations with the age of OCD onset. These findings seem to suggest the presence of an association between different symptom dimensions and leukocyte profiles, possibly related to certain inflammatory pathways. A lower platelet count was related to a sudden OCD onset, to the presence of past and current contamination obsessions, and to higher scores in the Y-BOCS items “insight” and “reliability.” As a matter of fact, thrombocytopenia might represent a common report in inflammation,^80^^–^^82^ and it is likely to constitute an important matter of investigation in these patients, given the crucial role exerted by platelets in OCD, in relation especially to serotonergic transmission and psychotropic drugs.^1^^,^^83^^,^^84^ Folate deficit was found in 20 out of 42 patients assessed for this vitamin. Folate levels were higher in women than in men, in agreement with literature data, and in those 30 subjects with no history of perinatal insults. Higher folate levels were associated with a lower “degree of control over obsessions,” that is to say, with a better control exerted on obsessive thoughts. The role of folate in OCD has been investigated in several studies, with controversial findings.^85^^–^^87^ According to the present study, higher folate levels might have a protective role against some symptomatology domains, although further studies are required to deepen this topic.

The vitamin B12 levels were within the normal range in 34, while a deficiency was present in 4 patients. However, interestingly, the higher the vitamin B12 levels, the higher the ASO titers, WBC and monocyte counts, and the higher the scores in “overvalued sense of responsibility” and “reliability” item scores. These findings seem to be opposite to the evidence of lower vitamin B12 levels reported in depressed and bipolar patients.^88^^–^^91^ In the current study, a vitamin B12 deficiency was assessed in 4 patients only. However, the significant correlations detected between vitamin levels and ASO titers and higher WBC and monocyte counts might suggest that it is involved in inflammation processes that are quite active in this subgroup of patients.

Vitamin D levels, albeit low, were not related to any clinical or laboratory parameters, except for the BMI: this is at variance with literature data showing vitamin D associated with overall severity and specific symptom patterns.^42^^,^^92^^,^^93^ In our opinion, this discrepancy might be due to the prevalent role of infections triggering and shaping both the biological patterns and the clinical features.

According to the available literature, immune system abnormalities are likely to play an important role in the pathophysiology of OCD.^10^^,^^43^^–^^45^ More specifically, an altered immune response to several kinds of noxae, such as stress and infections, seems to be involved in a complex pathophysiologic frame with interconnected genetic and environmental factors, although the exact mechanisms are yet to be fully explained. Indeed, it is conceivable that specific immune dysfunctions might lead to peculiar CNS alterations that would underlie different OCD clinical pictures and symptom dimensions. However, further evidence is needed to support this concept, as studies conducted to date are scant and often involve small samples of patients. Nonetheless, we are of the opinion that a deeper understanding of the immune mechanisms in OCD might pave the road to the development of novel treatment options.

A randomized trial conducted in children with recent-onset OCD and/or tics reported that cefdinir, a β-lactam antibiotic, improved, albeit not at statistically significant level both OCS and tics, with a good tolerability profile.^94^ The authors also suggested that the overall class of β-lactam antibiotics, beyond antimicrobial, might have neuroprotective properties.^94^ Intriguing findings have been reported for augmentation strategies with celecoxib, an NSAID belonging to the ciclooxigenase-2 (COX-2) selective inhibitors.^95^ Indeed, as used as an adjunctive treatment to fluoxetine, celecoxib provoked a more significant reduction in OCS than the SSRI plus placebo.^96^ Similar findings were also observed with the fluvoxamine-celecoxib combination.^97^

To date, antibiotic prophylaxis and administration of oral penicillin, immunotherapy, plasmapheresis, and intravenous immunoglobulins have been proposed as therapeutic options to reduce OCS, however, results remain controversial, especially in adults.^98^^,^^99^ However, a recent preliminary ongoing study of ours conducted in 20 subjects seems to convey encouraging results for therapeutic perspectives, as the augmentation strategy with antibiotics (one tablet a day for 2 days every 2 weeks for 3 months, as recommended by the guidelines for Streptococcus eradication), associated with one SRI or SSRI, led to a significant decrease in the Y-BOCS score, as assessed at the follow-up after 3 and 6 months (data not shown).

The small size of the sample is the main limitation of this study, and, although all patients were well characterized from the clinical point of view, a larger sample might lead to more specific findings. Another problem is the absence of a control group for a further comparison, as we are planning to do in the future. Indeed, further studies assessing not only the characteristics of ASO titer-positive OCD subjects who are asymptomatic for pharyngotonsillitis but also the differences with ASO-seronegative OCD patients are warranted to better disentangle this intriguing matter.

## Conclusions

Taken together, the findings of the present study indicate that about 25% of OCD patients, despite having no past or current symptoms of bacterial infections, may show ASO titer-positivity together with alterations of some peripheral markers, suggestive of a chronic inflammatory state. Therefore, past and silent streptococcal infections cannot be considered so “silent” but dangerous at least in a subgroup of patients, especially if males and with a history of perinatal traumas.

According to us, the evaluation of streptococcal infections and of peripheral biomarkers should be included in routine assessment of OCD patients, irrespective of age and clinical picture, given their ease and low cost. As OCD is a chronic psychopathological condition with a high rate of treatment resistance, our and other studies suggest that some patients might benefit from the administration of anti-inflammatory drugs and antibiotics targeting the GABHS (and possibly other) infections that might be involved in the onset and the maintenance of OCD and related disorders. In addition, our data seem to support the notion of a certain anti-inflammatory activity of some psychotropic compounds, a topic that strongly requires being deepened in future studies.
